# HydroGuard: a controlled release hydrogel-phage delivery system to combat catheter-associated urinary tract infections

**DOI:** 10.3389/fsysb.2026.1923468

**Published:** 2026-10-22

**Authors:** Shilpa Rao, Rhyan McLean, Aadila Zakir Hussain

**Affiliations:** 1 Waterloo iGEM 2025, Faculty of Engineering, University of Waterloo, Waterloo, ON, Canada; 2 Waterloo iGEM 2025, Faculty of Science, University of Waterloo, Waterloo, ON, Canada

**Keywords:** antimicrobial resistance, bacteriophage therapy, catheter-associated urinary tract infections, computational modelling, hydrogels

## Abstract

Catheter-associated urinary tract infections (CAUTIs) rank as the fourth-most significant nosocomial infection globally. CAUTIs account for approximately 80% of hospital-acquired urinary tract infections and represent nearly one-third of all device-associated infections. Urinary catheters serve as a hub for bacteria to form highly resistant biofilms containing pathogens, like *Enterococcus* (E.) faecalis that reduce effectiveness of the immune system and antibiotic treatments. Therefore, tackling biofilm formation at the catheter surface becomes a crucial step in mitigating CAUTIs. We aim to address *E. faecalis* biofilm formation by engineering HydroGuard: a Gelatin Methacryloyl (Gel-MA)-based hydrogel injected with bacteriophages, to provide targeted, enzyme-triggered antimicrobial activity. *Enterococcus faecalis* expresses gelatinase (GelE), a zinc metalloprotease that enhances biofilm formation. GelMA is susceptible to GelE-mediated degradation, enabling enzyme-triggered release of bacteriophages that specifically lyse *E. faecalis*. For a Biosafety Level 1 proof-of-concept experiment with a well-studied host and phage system, we heterologously expressed GelE in *Escherichia* (E.) coli DH5α to emulate native *E. faecalis* expression. This system was tested on dynamically grown biofilms, to simulate similar conditions to the urinary tract. Currently, the *E. coli* system has shown preliminary inhibition when tested against the hydrogel product, however testing of different phage combinations is needed. Mathematical modelling identified hydrogel crosslinking density as a critical tuneable parameter governing therapeutic availability and predicted nonlinear, degradation-dependent phage release profiles. Sensitivity analyses revealed design trade-offs between structural stability and antimicrobial responsiveness. Together, the biofilm, hydrogel, phage-bacteria, and protein design models form a multiscale computational framework that links biofilm development and environmental conditions to enzyme-mediated hydrogel degradation, phage release, and bacterial suppression. Agent-based biofilm simulations captured emergent spatial dynamics under nutrient, flow, and pH constraints, while a coupled predator-prey model investigated phage-bacteria population dynamics within the degrading GelMA matrix. Computational protein design further explored collagen-like peptide motifs with enhanced folding stability and preserved gelatinase cleavage sites, providing a molecular-level approach to tuneable degradation kinetics. This multiscale framework provides predictive insight into how biofilm conditions, hydrogel degradation, and phage availability interact within the proposed system, while the preliminary experimental observations provide supporting evidence for its feasibility. By integrating synthetic biology, tissue engineering, and modelling, HydroGuard offers a novel, non-antibiotic strategy for CAUTI prevention.

## 1 Introduction

Urinary tract infections (UTIs) are infections affecting any part of the urinary system, and are among the most common bacterial infections worldwide, affecting 150 million people annually (Stamm and Norrby, 2001). The risk of acquiring a complicated infection is significantly heightened for patients who require long-term urinary catheterization, with the likelihood increasing by 3%–7% every day post-placement (Gould et al., 2010). In the United States, the CDC reported that catheter-associated urinary tract infections (CAUTIs) account for 12.9% of all hospital-acquired infections, representing a major public health and economic burden (Magill et al., 2014).

The pathogenesis of CAUTIs arises from the ability of microorganisms to bypass the body’s natural defense mechanisms through the catheter, adhering to surfaces and forming biofilms within the urinary tract (Werneburg, 2020). Biofilms are complex microbial communities encased in an extracellular matrix that enhances bacterial survival and antimicrobial resistance (AMR). Further complicating CAUTI treatment is the prevalence of polymicrobial infections, consisting of two or more bacterial species co-existing on the catheter. These infectious biofilms often develop in patients within 1–3 days of catheterization (Gunardi et al., 2021). Once established, they are difficult to eradicate and can result in recurrent or persistent infections that contribute to patient morbidity and mortality (Codelia-Anjum et al., 2023).

The most common microorganisms involved in CAUTIs include *Escherichia coli*, *Enterococcus*, *Klebsiella* spp., *Pseudomonas aeruginosa*, and *Staphylococcus aureus* (Oumer et al., 2021). The *Enterococcus* species, mainly *Enterococcus faecalis* and *Enterococcus faecium*, account for 15%–30% of CAUTIs (Guiton et al., 2010) due to their mechanisms that enhance their pathogenicity. Among existing strategies for addressing catheter-associated biofilms, antimicrobial coatings and hydrogels can provide localized bacterial inhibition, while metal alloy-coated catheters primarily target bacterial adhesion. However, these approaches generally rely on continuous or passive antimicrobial activity rather than a response to the presence of a biofilm-associated signal.

*Enterococcus faecalis* is a Gram-positive, facultative anaerobic bacterium that thrives in the catheterized urinary tract environment. It adheres to catheter surfaces, forming biofilms and producing a zinc metalloproteinase called GelE (Del Papa et al., 2007). GelE degrades host proteins such as fibrinogen (which accumulates on the catheter due to the potential inflammation of the uroepithelium upon catheterization) into peptides, which bacteria utilize for nutrition, while simultaneously promoting biofilm growth and persistence (Codelia-Anjum et al., 2023; Ch’ng et al., 2019). Furthermore, *E. faecalis* has strong AMR capabilities, often necessitating repeated or prolonged courses of antibiotics (Del Papa et al., 2007). These properties make *E. faecalis* a critical pathogen to target in CAUTIs.

Current clinical strategies to mitigate CAUTIs focus primarily on improved hygiene, catheter management protocols, and antibiotics. These approaches do not eliminate the underlying issue of biofilm formation, and infections remain common, particularly among elderly, critically ill, and chronically catheterized patients who are likely to repeatedly contract these infections (CDC, 2025). As a result, CAUTIs continue to strain global healthcare systems by causing significant patient discomfort, hospital readmissions, and clinical complications (Maharjan et al., 2018; Zhang et al., 2019).

Catheter-integrated, preventative strategies such as metal alloy-coated catheters, antimicrobial hydrogels, and phage-based therapy for reducing biofilm formation have emerged as promising alternatives to antibiotic treatment for complicated UTIs (Aljohi et al., 2016; Kai-Larsen et al., 2021; Zalewska-Piątek and Piątek, 2020). While these approaches demonstrate the potential of localized antimicrobial delivery, conventional coatings and hydrogels generally provide antimicrobial activity through passive or sustained release. Smart, stimuli-responsive hydrogels use local environmental cues to control therapeutic release, providing an opportunity to couple antimicrobial delivery to infection-associated conditions.

This study presents a novel, GelMA hydrogel system embedded with a bacteriophage (phage) cocktail, designed to release phages in response to GelE-mediated degradation and directly target *E*. *faecalis* biofilms on catheter surfaces. GelMA is a biocompatible and biodegradable polymer derived from gelatin, easily functionalized via ultraviolet (UV) photocrosslinking (Sun et al., 2018; Dorterler et al., 2024; Zhao et al., 2016). Unlike conventional antimicrobial coatings, this design uses the local presence of a biofilm-associated enzyme to trigger phage release, providing a stimulus-responsive mechanism for targeted antimicrobial delivery. Future investigations should consider the expansion of this bioresponsive phage-hydrogel system as treatments for other biofilm-forming bacterial strains implicated in CAUTIs.

Computational modelling of biological systems enables investigation of emergent behaviours like biofilm growth, enzymatic degradation, and spatiotemporal drug release that are difficult to resolve experimentally. These modelling approaches aim to capture complementary stages of the same therapeutic system, linking together biofilm development, hydrogel degradation, and phage release.

In the context of CAUTIs, computational modelling offers a way to explore how factors like flow, nutrient gradients, enzyme secretion, and material properties collectively influence infection dynamics at the catheter surface. Model parameters were obtained from published literature and experimental results where available, with sensitivity analyses performed for models containing uncertain biological parameters. The resulting models were used together to examine how changes in the biofilm environment could influence hydrogel degradation and therapeutic availability, and how these changes could subsequently affect phage-bacteria dynamics.

By integrating computational modelling with experimental design, this work seeks not only to evaluate the feasibility of this system, but also to establish a generalizable framework for engineering enzyme-responsive antimicrobial biomaterials.

## 2 Materials and methods

The methodology is centred around three interconnected components: GelE production and characterization, GelMA hydrogel fabrication, and phage encapsulation and release. Alongside these experimental components, computational modelling was used to examine biofilm development, hydrogel degradation, phage release and phage-bacteria interactions as part of the same system.

### 2.1 Cloning

For testing under BSL-1 conditions in accordance with iGEM safety requirements, DH5α was engineered to express GelE using the commercially available plasmid pUC19. Both pUC19 and primers targeting the GelE gene were purchased from Integrated DNA Technologies Inc. All primers were designed using the SnapGene primer design feature.

The use of *E. coli* as the expression host provided a Biosafety Level 1 model for evaluating the feasibility of enzyme-responsive hydrogel degradation, while avoiding direct handling of the pathogenic target organism. Although this system enables evaluation of GelE expression, it does not fully replicate the physiology, regulation of gelE expression, or biofilm behaviour of native *E. faecalis*. Consequently, results obtained using this surrogate system should be interpreted as preliminary and as representative of the surrogate system instead of native *E. faecalis*.

The primers designed for amplification of the pUC19 vector and gelE fragment, respectively:

PUC19_F: CTG​GTC​AAC​GAA​TAA​ACT​GGC​CGT​CGT​TTT​AC

PUC19_R: TTA​CCC​TTC​ATG​GCG​TAA​TCA​TGG​TCA​TAG​C

gelE_F: GAC​CAT​GAT​TAC​GCC​ATG​AAG​GGT​AAC​AAA​ATT​CTG​TAC

gelE_R: CGA​CGG​CCA​GTT​TAT​TCG​TTG​ACC​AGC

QIAGEN’s QIAquick Gel Extraction kit was used to purify the cloned fragments.

The GelE gene was integrated into the pUC19 vector using a Gibson Assembly protocol adapted from a student advisor, Nicole LeBlanc. 10µL reactions were prepared including 25 ng of both the fragment and plasmid, PCR water, and 5 µL of Hifi Gibson Mastermix. Samples were incubated in the thermocycler for 1 h at 50 °C.

Post Gibson Assembly, pUC19-gelE was transformed into DH5α competent cells using heat shock and successful colonies were verified using PCR. An overnight culture of the DH5α with pUC19-gelE was prepared with a transformed colony, then miniprepped using the QIAGEN miniprep kit. The miniprepped sample was then heat shocked into BL21 competent cells for optimized protein expression. These transformation plates were used to make another overnight culture, which was then saved as a freezer stock with 7% DMSO in Luria-Bertani (LB). This freezer stock contained the final PUC19 with GelE in the *E*. *coli* strain BL21.

### 2.2 Biofilm simulation

BL21 *E. coli* biofilms were grown for 3 days on glass microscope slides at 37 °C, with continuous shaking, and replenishing of LB media daily. This mimicked a dynamic environment with shear stress and was intended to replicate the catheter environment. The methods for biofilm growth were based off of studies conducted by Sternberg et al., who explored three dynamic models for biofilm formation (Sternberg et al., 2014).

This experimental system was designed as a prototype rather than a direct representation of catheter-associated *E. faecalis* biofilms and their behaviour. Accordingly, observations from this model were used to assess the feasibility of the HydroGuard concept and were interpreted alongside computational modelling instead of definitive evidence of clinical performance.

### 2.3 Hydrogel fabrication and characterization

The GelMA solution was created by dissolving gelatin in 1X PBS solution until dissolution. Methacrylic anhydride (Sigma Aldrich) was then added dropwise to the solution while stirring at 50 °C, for approximately 1 h. The solution was then quenched with additional 1X PBS. To remove excess methacrylic anhydride, the solution was first pelleted by centrifugation at 1000 x g for 2 min and supernatant is collected. Supernatant is then dialyzed in DI water to remove PBS salt for 72 h at 40 °C, exchanging water every 24 h. The dialysis tubing used was purchased from Flinn Scientific and has a molecular weight cutoff of 12,000–16,000 Da. For long-term storage, this solution was lyophilized for 6 days.

Freeze dried aliquots were used to prepare fresh batches of hydrogel when needed. From preliminary experimentation, the optimal GelMA concentration was found to be 15% (w/v) based on texture and flexibility of the gel. An optimal concentration of the photoinitiator, Irgacure (Sigma Aldrich), was found to be 0.5% based on curing efficiency. To form the hydrogel, lyophilized GelMA was dissolved at 40 °C in 1X PBS, after which photoinitiator was added. Hydrogels were then cured for approximately 25 min under a 254 nm lamp (germicidal wavelength). Hydrogels were formed in a 6 × 6 cm plastic container with a height of 1 cm.

Hydrogel composition was optimized based on preliminary laboratory observations of curing efficiency, structural integrity, and handling characteristics during fabrication. Comprehensive material characterization, including swelling behaviour, pore morphology, rheological properties, and microstructural imaging, was beyond the scope of the present concept validation and will be required in future work to further evaluate the hydrogel for biomaterial applications.

### 2.4 Bacteriophage cocktail design

Whitelisted *E. coli* phages were donated to the team by the Maxwell Laboratory at the University of Toronto. A lytic phage cocktail comprising T7, T4, T2, and λ was selected for incorporation into the hydrogel matrix. These phages were chosen for their broad host range and demonstrated activity against biofilm-forming bacteria. Propagation was done to increase concentration, by adding 5 mL of LB, 200 µL of λpir O/N (a donor strain), and 5 µL of the phage stock to a tube, and incubated at 37 °C. These cells were pelleted by centrifuging for 3 min at maximum RCF, and then filter sterilized with a 5 mL syringe and 25 mm PES filter. To conduct the phage spotting assay, the double overlay technique was used. Serial dilutions 1:10 were prepared with LB in a 96-well plate. 3 mL of LB and 150 µL of the λpir O/N were added to a tube and stored at 55 °C, then swirled on an LB thick plate to form a lawn. The lawns were left to dry for 5 min, and then 2 µL of each diluted phage was spotted and incubated at 37 °C for 3 days.

Steps 2 and 3 of the phage extraction protocol from the Center of Phage technology were used.

To perform genome digestions, the following RE digests were setup:

**Table:** 

Phage	RE	Phage volume (µL)
λvir	EcoRI	8
T2	SspI	4
T4	PacI	4
T7	BsmI	4

These digests were set up in 1.5 mL microcentrifuge tubes along with 1 µL of rCutSmart. 1 μL of each restriction enzyme (RE) was added. These digests were observed on a 1% agarose gel ran at 30 V.

### 2.5 Testing of HydroGuard against biofilm

Hydrogels were prepared and tested in 20 mL batches. A sterile syringe was used to inject 100 µL of phage mixture slightly underneath the surface of the hydrogel, in the original container it was cured in. Once mature biofilms formed after the 72-h growth period, slides were removed from the 6 × 6cm plastic container and placed biofilm side down onto the hydrogel.

This system was placed at 4 °C for 24 h to prevent phage death. After 24 h, 10 µL of biofilm liquid exposed to the hydrogel around the phage injection site was collected. Dilutions (10^–2^, 10^–4^, 10^–6^, and 10^–8^), were plated on LB agar ampicillin100 plates and grown at 37 °C for 44 h. For comparison, a biofilm exposed to a hydrogel not injected with phage was also plated and incubated for 44 h, as a negative control.

### 2.6 Computational modelling

Computational analyses were performed in parallel with experimental work to characterize biofilm dynamics, hydrogel degradation, phage release, and phage-bacteria population interactions. All simulations were implemented in Python using numerical solvers for ordinary (O) and partial (P) differential equations (DE).

The computational framework was developed to investigate system behaviour that could not be directly measured within the scope of the present experiments. Established mathematical models from the literature were selected according to the biological process being represented, allowing individual components of the HydroGuard system to be examined before integrating them into a multiscale framework. The biofilm model describes the environment in which GelE is produced, the hydrogel model describes enzyme-mediated degradation and phage release, and the phage-bacteria model describes the resulting interaction between released phages and bacteria. Wherever possible, parameter values were obtained from published experimental studies and preliminary in-lab work, while parameters unavailable for the target system were estimated from biologically plausible literature ranges and evaluated through sensitivity analyses.

To model CAUTI biofilm formation, *E. faecalis* colonization was represented across three biological phases: attachment, spread, and detachment. Conditioning film attachment was described using a Langmuir adsorption model, simulating how urinary proteins coat the catheter surface to provide binding sites for bacteria. Bacterial transport to the surface was modelled with an advection-diffusion framework, and irreversible surface attachment was captured using Smoluchowski kinetics.

Biofilm spread along the catheter was simulated using a Fisher-Kolmogorov-Petrovsky-Piskunov (FKPP) reaction-diffusion model. Two coupled variables, surface biomass density (CFU/cm^2^) and biofilm thickness (µm), were tracked over time and space, enabling the conversion of lateral growth into vertical accumulation. Surface biomass density dictated the rate of detachment, which was modelled as the sum of erosion and periodic sloughing events using an ordinary differential equation framework. The erosion rate was dependent on biofilm density and extracellular polymeric substances (EPS) fraction, while sloughing frequency was tied to the surface organic loading rate (SOLR). Parameters for attachment, spread, and detachment were drawn from literature and previous experimental studies (Bull et al., 2025; Pechaud et al., 2024). These models were selected because they describe complementary stages of biofilm formation and have previously been applied to catheter-associated biofilm systems. Their integration enabled prediction of biofilm development across multiple spatial and temporal scales while remaining consistent with published biological behaviour. The resulting biofilm state provides the environmental context for the downstream hydrogel degradation and phage-release models.

GelMA hydrogel degradation and phage diffusion were simulated using a PDE framework. GelMA breakdown was parameterized as a function of GelE concentration, resulting in time-dependent changes in hydrogel geometry and effective diffusivity. Phage transport through the matrix was modelled as diffusion with enzyme-dependent release kinetics. Immobilized phages transitioned to mobile phages according to first-order kinetics, and boundary conditions representing an infinite sink were applied at the hydrogel surface. The diffusion framework was selected because diffusion is expected to be the dominant transport mechanism within hydrated GelMA matrices. Model outputs therefore represent predicted release behaviour based on the assumed degradation kinetics rather than experimentally validated release profiles.

Bacterial-phage population dynamics were captured with a modified Lotka-Volterra predator-prey model. Key parameters included phage adsorption rate, latent period, burst size, phage decay rate, and bacterial growth rate. *Escherichia coli* kinetic parameters were obtained from literature, while *E. faecalis* parameters were sampled across biologically plausible ranges using Monte Carlo sensitivity analysis with 1,000 simulations. Sensitivity analysis was used to evaluate the robustness of model predictions across uncertainty in biological parameters, particularly where experimentally measured values for *E. faecalis*-specific phage interactions were unavailable.

To simulate controlled phage availability, the predator-prey model was coupled to the hydrogel degradation model. Unlike classical Lotka-Volterra formulations that assume immediate phage availability, this coupled system incorporated time-dependent phage release driven by enzyme-mediated matrix degradation so that bacterial suppression was modelled under the release conditions predicted by the hydrogel model. A total of 500 simulations were performed across varying initial bacterial and phage concentrations. Outputs included bacterial population trajectories and time to suppression below the defined infection threshold.

Collagen-like peptide sequences for hydrogel reinforcement were optimized while preserving GelE-responsive degradation. Variants were generated using a message-passing neural network (ProteinMPNN) framework constrained to collagen-like repeat motifs. Structural predictions were obtained using AlphaFold3 WebServer to assess folding confidence and integrity. Candidate sequences were refined using PyRosetta for energy minimization and structural validation. Sequences were filtered based on predicted folding confidence, similarity to parent motifs, and preservation of accessible gelatinase cleavage sites. ProteinMPNN, AlphaFold3, and PyRosetta were selected because they provide complementary capabilities for sequence generation, structural prediction, and energetic evaluation, respectively, allowing candidate collagen-like peptides to be screened computationally before experimental implementation within the same framework used to evaluate hydrogel performance.

Integrating the computational models with preliminary wet-lab experimentation suggests that the proposed HydroGuard system can support enzyme-responsive phage release as intended. The diffusion model predicts effective transport of phages through the degrading hydrogel matrix, while the preliminary experimental observations support the feasibility of the overall design. However, experimental validation of phage release kinetics and diffusion remains necessary to confirm these computational predictions.

## 3 Results

### 3.1 Phage propagation and validation

Four *E. coli* phages (T2, T4, T7 and, λvir) were successfully propagated and tested for lytic activity. Clear lysis zones on *E. coli* lawns were observed, confirming that all phages retained infectivity under the used experimental conditions (Figure 1).

**FIGURE 1:**
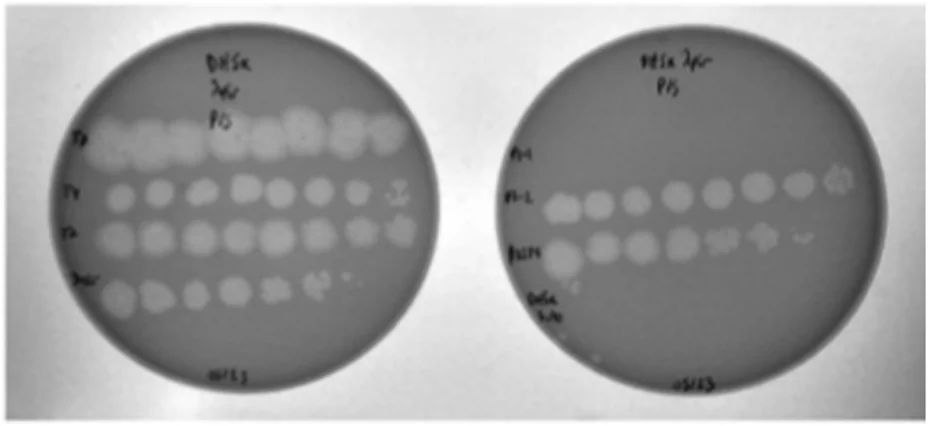
Genome Extractions of Titred E. coli Phages T2, T4, T7, and, λvir.

Agarose gel electrophoresis of extracted genomic DNA revealed strong bands for T2, T4, and T7, while λvir yielded faint or degraded bands. Restriction digestion produced distinct banding patterns for T2, T4, T7, and that matched *in silico* predictions (Figure 2). λvir digestion produced weak but interpretable fragments. Additionally, pUC19 and GelE were successfully amplified and extracted (Figure 3). Qualitative observations indicated differences in bacterial growth following exposure to hydrogels containing different hage formulations (Figure 4).

**FIGURE 2:**
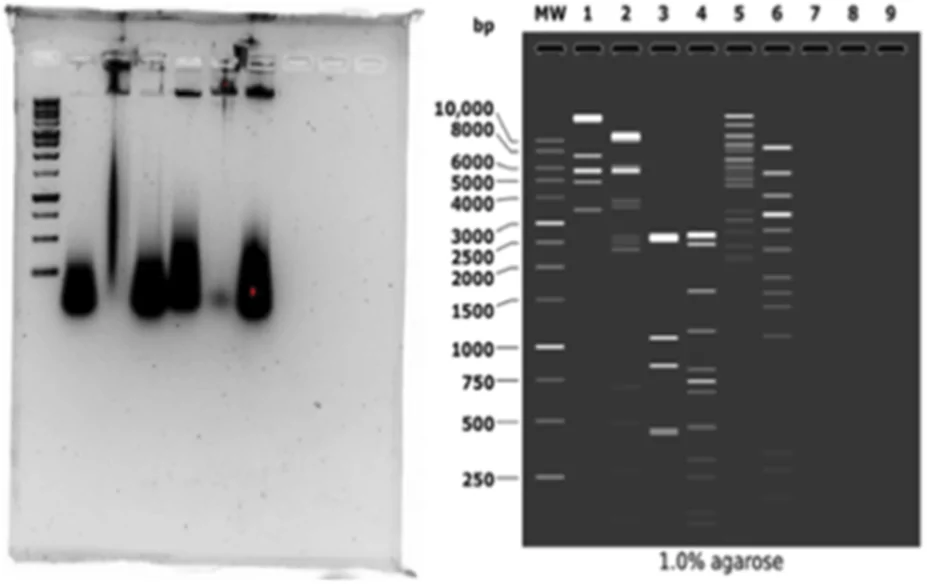
Gel Electrophoresis of Extracted Genome Digestions and Simulated Gel.

**FIGURE 3:**
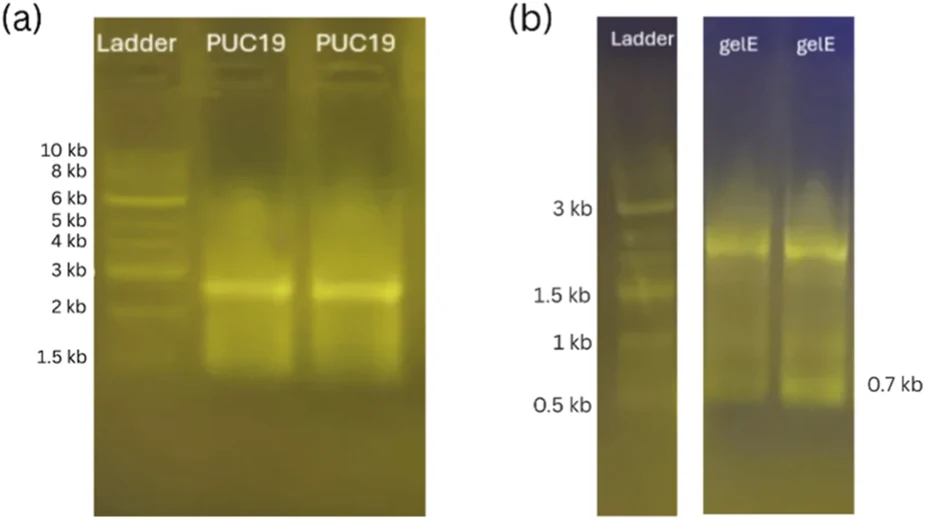
Plasmid Validation for GelE Expression **(a)** Gel visualization of amplified pUC19. A 1 kb Quick-load ladder was used. pUC19 has a size of 2.7 kb. **(b)** Gel visualization of amplified gelE using a 1 kb Quick-load ladder.

**FIGURE 4:**
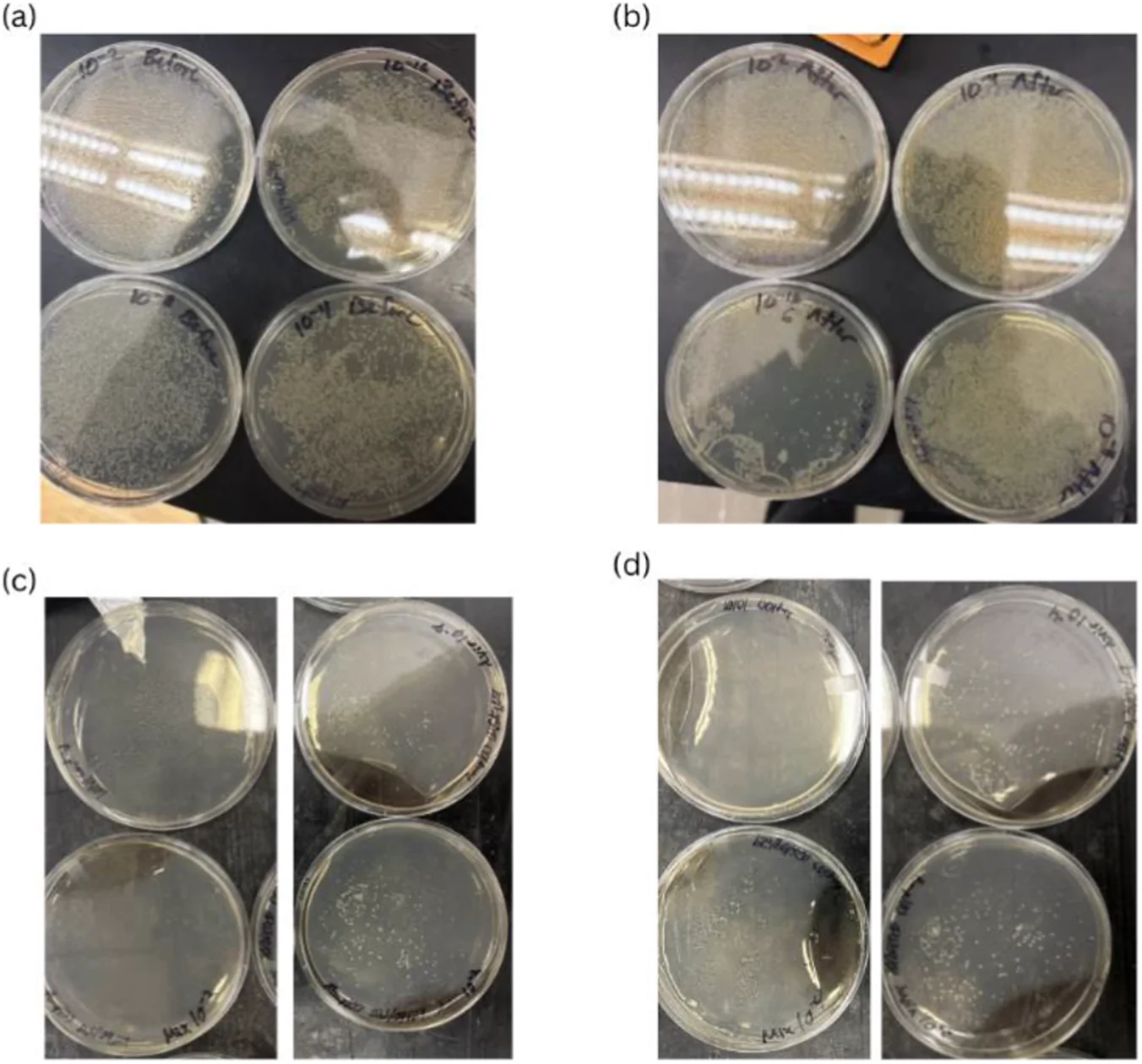
**(a)** Negative control: 10^–2^, 10^–4^, 10^–8^, 10^–16^ dilutions of biofilm collected 44 h after incubation on the hydrogel, not exposed to phages **(b)** Dilutions of biofilm collected after 44-h exposure to hydrogel injected with T7 phage **(c)** Exposure plates after 44 h of exposure to hydrogel injected with λvir **(d)** Exposure plates after 44 h of exposure to hydrogel injected with the phage mixture (T4, T7, *λ*vir).

### 3.2 Testing of HydroGuard against biofilm

Qualitative observations indicated differences in bacterial growth following exposure to hydrogels containing different phage formulations. Among the conditions tested, the phage cocktail produced the greatest apparent reduction in visible bacterial growth, whereas individual phages produced more limited inhibition. As these observations were acquired from a proof-of-concept experiment without quantitative bacterial enumeration or statistical analysis, they should be interpreted as preliminary evidence supporting the feasibility of the HydroGuard concept rather than definitive measures of antimicrobial efficacy.

### 3.3 Model of *E. faecalis* biofilm formation

As biofilm formation consists of several unique stages, this model was developed by joining together and modifying parts of models from external sources (Bull et al., 2025; Pechaud et al., 2024). Overall, it was simplified into three general stages to represent biofilm formation: attachment, spread, and detachment. This simplification was made to balance biological realism with computational tractability.

One of the outputs of the attachment model is the cell attachment rate per second. This value is designed to inform the growth rate that is used in the model representing the spread of the *E. faecalis* on the catheter. Another output describes the time it takes to reach 30% coverage of the catheter with the conditioning film, which is a critical point in the biofilm growth (Alotaibi and Bukhari, 2021). This model predicts the average and maximum cell density at the end of the simulation time, which is 1 h in this simulation. Figure 5 below illustrates representative outputs generated by the computational model.

**FIGURE 5:**
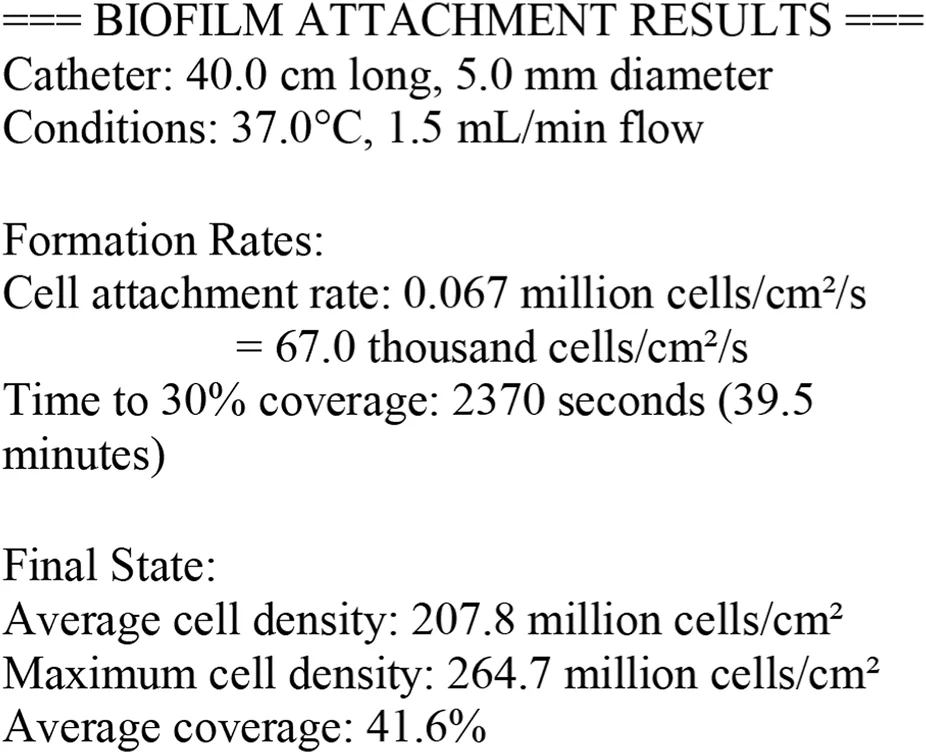
The attachment model determines the growth rate of the conditioning film of the biofilm.

The biofilm spread model focused on the spatial density of the biofilm over time, while also considering thickness. Figure 6 depicts the output of the model, biofilm density (*u(x,t))* along the length of the catheter, with each curve depicting the spatial profile at a different point in time.

**FIGURE 6:**
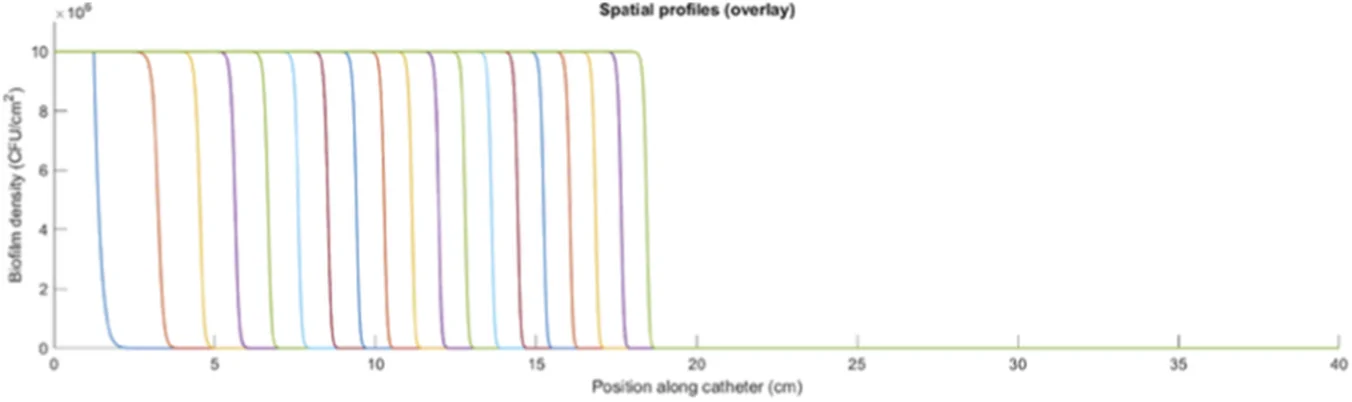
Spatial profile of biofilm density along the catheter length over time.

The output of the biofilm detachment model is the change in biofilm thickness over time, both from growth calculated from the results of the attachment model, surface erosion, and sloughing events. Figure 7 plots the biofilm thickness at different time steps over the 35 h period modelled. These simulations define the biofilm environment in which the downstream hydrogel and phage models are considered.

**FIGURE 7:**
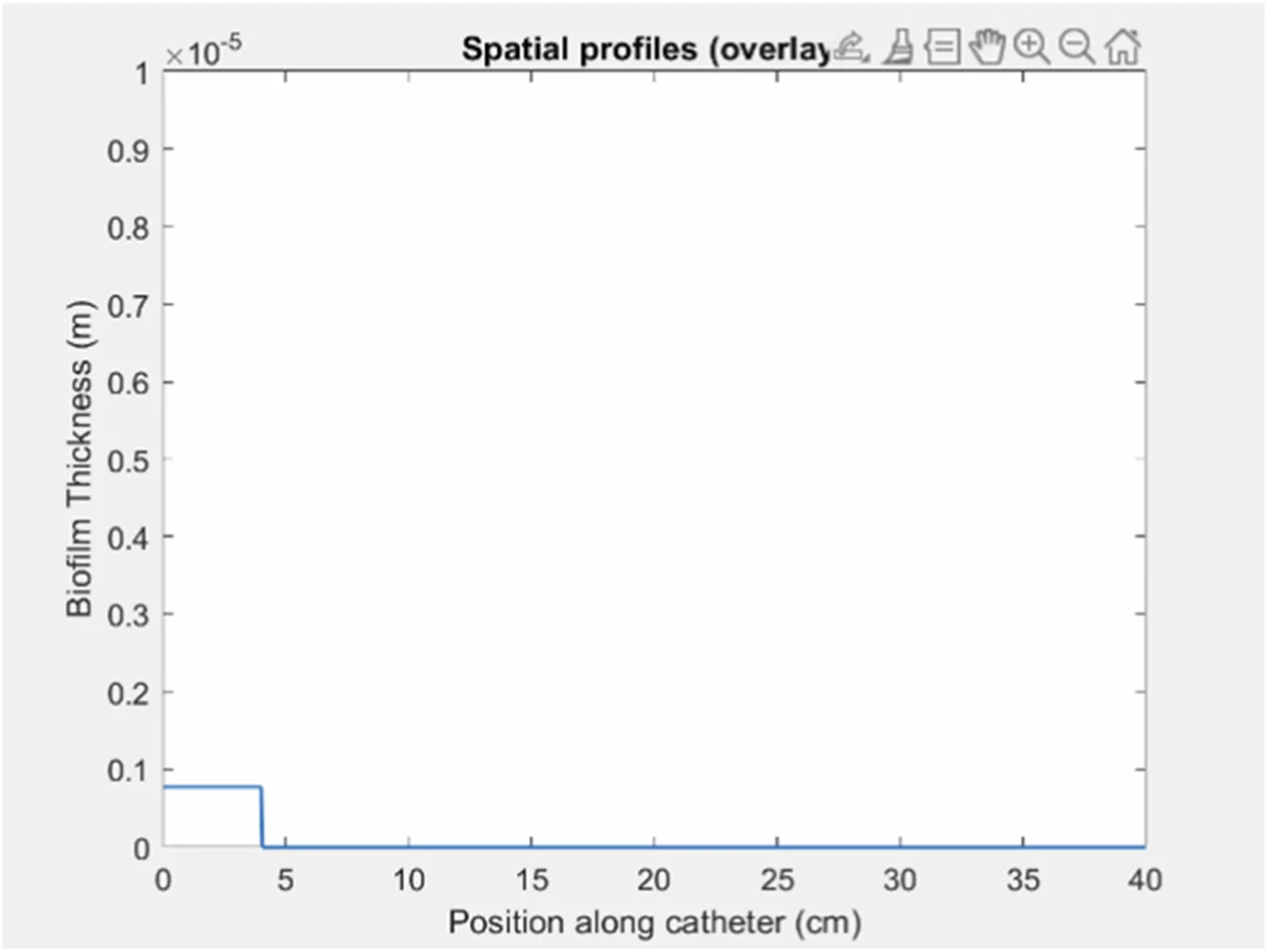
Biofilm thickness plotted along length of catheter over time.

### 3.4 Hydrogel diffusion modelling

A computational diffusion model was developed to predict the release of bacteriophages from crosslinked gelatin-based hydrogels under varying concentrations of GelE.

The simulation predicted that cumulative phage release would reach approximately 10% after ten hours under the simulated conditions (Figure 8). The behaviours of the immobilized phage. The behaviours of the immobilized phage, mobilized phage, and the released phage were also reproduced (Figure 9). The model generated cumulative release curves showing biphasic release behaviour. An initial burst release occurred due to the diffusion of mobile phage where the mobile phage increases as immobilized phage transitions into a mobile state within the hydrogel, followed by a slower sustained release phase corresponding to GelE-mediated degradation of the matrix. The model predicted increasing release rates with higher enzyme concentrations.

**FIGURE 8:**
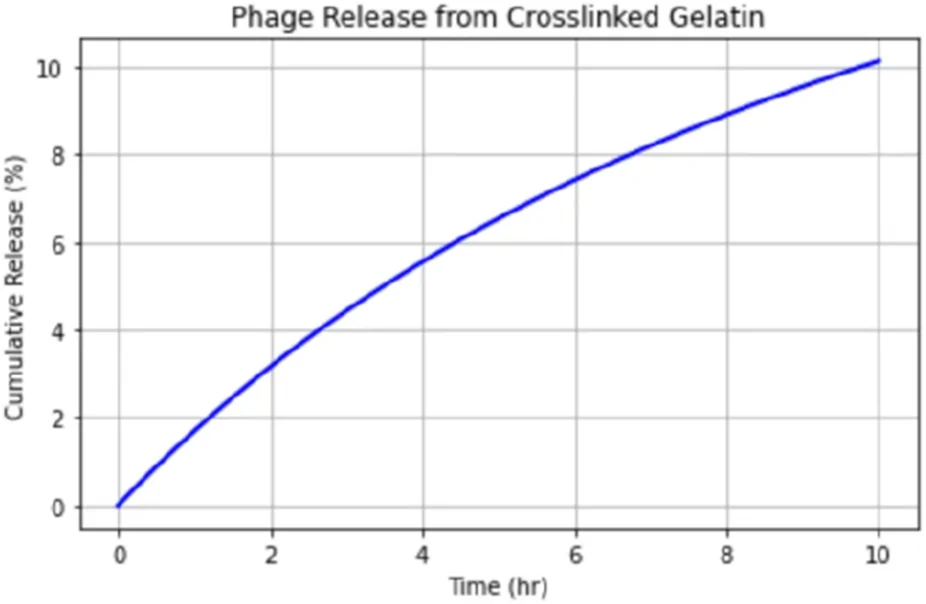
Computationally predicted phage release from a crosslinked GelMA hydrogel over time under the simulated conditions.

**FIGURE 9:**
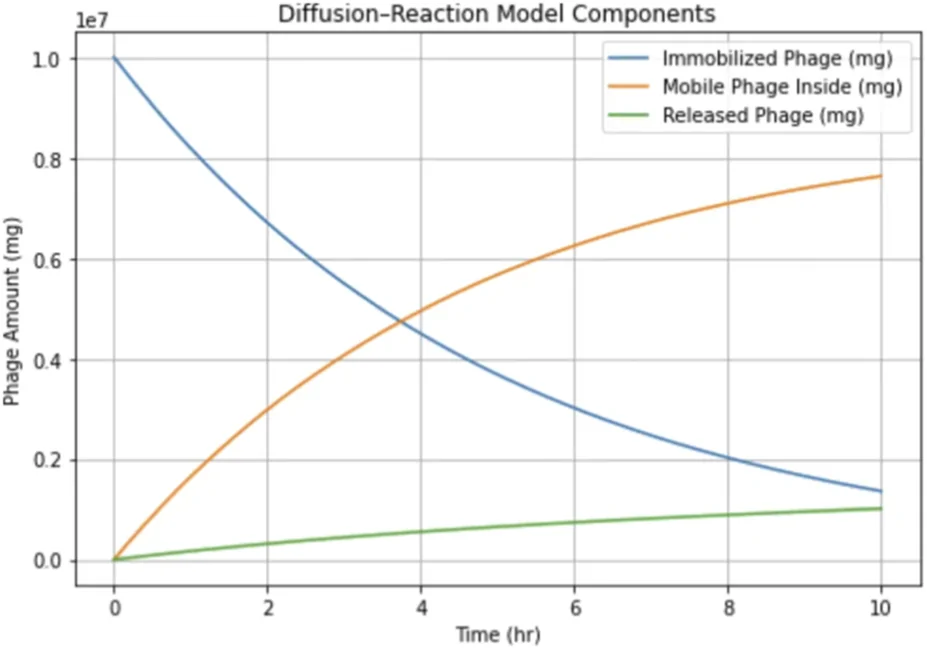
Diffusion-reaction models of the amount of phage (mg) over the course of hours where the blue line depicts the immobilized phage; the orange phase represents the mobile phage inside the hydrogel structure (mg); the release from within the hydrogel of the phage (mg).

These predicted release profiles have not yet been experimentally validated and should therefore be interpreted as computational projections intended to guide future experimental optimization.

### 3.5 Modelling the phage-bacteria interaction

Parameters such as phage adsorption rate, latent period, burst size, phage decay rate, and bacterial growth rate under optimal conditions were incorporated into the model for each phage-bacteria interaction. Due to the extensive characterization of *E. coli* phages in the literature, kinetic parameters for this system were more readily available and thus the results more realistically model the interaction. For *E. faecalis* phages, many of these parameters were not directly available during the experimental phase. Therefore, biologically plausible parameter ranges were sampled using a Monte Carlo sensitivity analysis with 1,000 simulations to identify parameter sets that maximize phage infectivity and bacterial suppression. Initial conditions for bacterial and phage populations were determined based on the phage loading within the hydrogel and reported bacterial concentrations in urine from previous research, which are typically near zero in normal urine environments. A defined threshold concentration was used to represent clinically relevant bacterial levels associated with infection or biofilm formation. Model results predict that bacterial concentrations remain above this threshold for less than 10 h, suggesting the potential for reduced likelihood of infection establishment under the simulated conditions (Figure 10).

**FIGURE 10:**
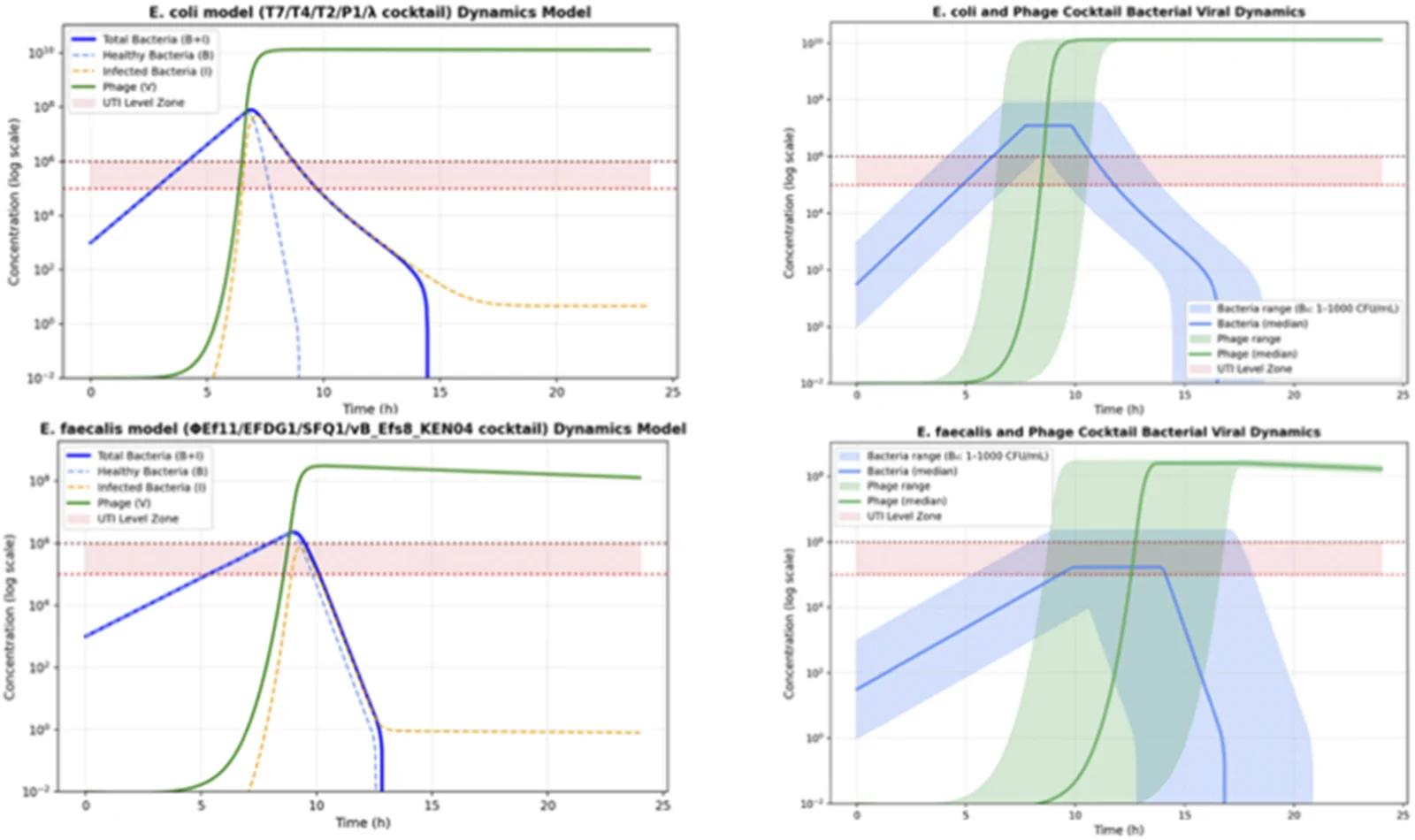
Representative outputs from the modified Lotka-Volterra computational model describing predicted interactions between selected bacteriophage cocktails and bacterial hosts.

To examine how the predicted phage availability translated into bacterial suppression, 500 simulations were performed across varying initial bacterial and phage concentrations, generating three sets of heatmaps: bacterial log reduction, time required to suppress bacterial levels below the danger threshold, and an overall treatment success score derived from these metrics. These heatmaps were used to identify parameter regions associated with consistent treatment success, with candidate experimental concentration ranges selected from regions exhibiting favourable outcomes across all three analyses (Figure 11).

**FIGURE 11:**
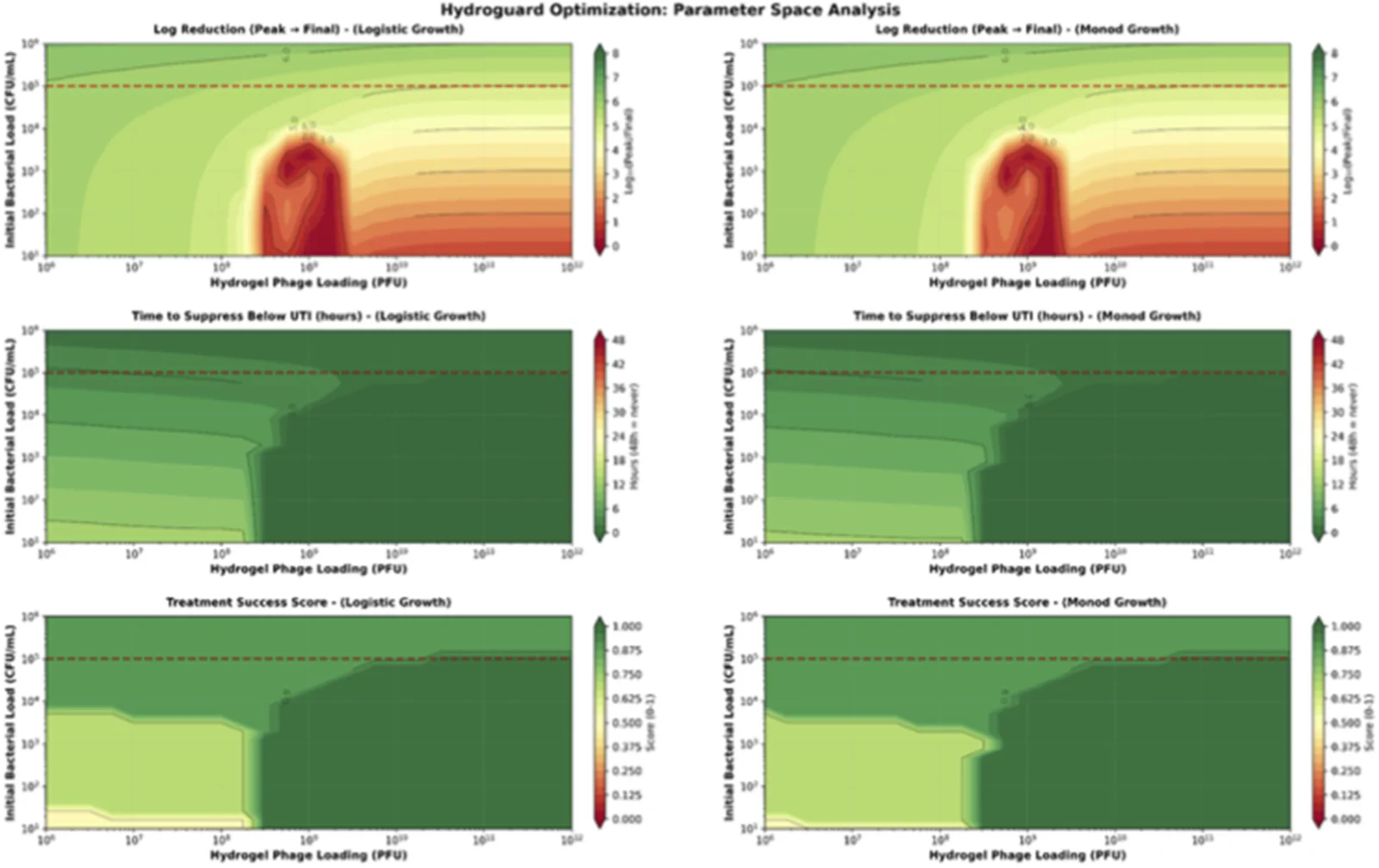
Computational sensitivity analysis identifying candidate concentration ranges associated with favourable simulated treatment outcomes.

### 3.6 Protein design modelling

A series of key steps were followed in generating viable GelMA subunits to propose a synthetic hydrogel, including Structural Determination of Collagen-Like Subunits, Generation of New Sub-Unit Sequences, Prediction of Structure of Designed Sub-Unit Sequences, and Quantification of Cross-Linking and Cleavage Sites.

Within the computational design framework, folding repeated GPH subunits always resulted in high-confidence outputs, where the plDDT, as predicted by AlphaFold3, was always greater than 90. Moreover, the folded models maintained the helical structure that was expected of such collagen-like sequences. A sample resultant visualization, created in PYMOL, is pictured in Figure 12.

**FIGURE 12:**
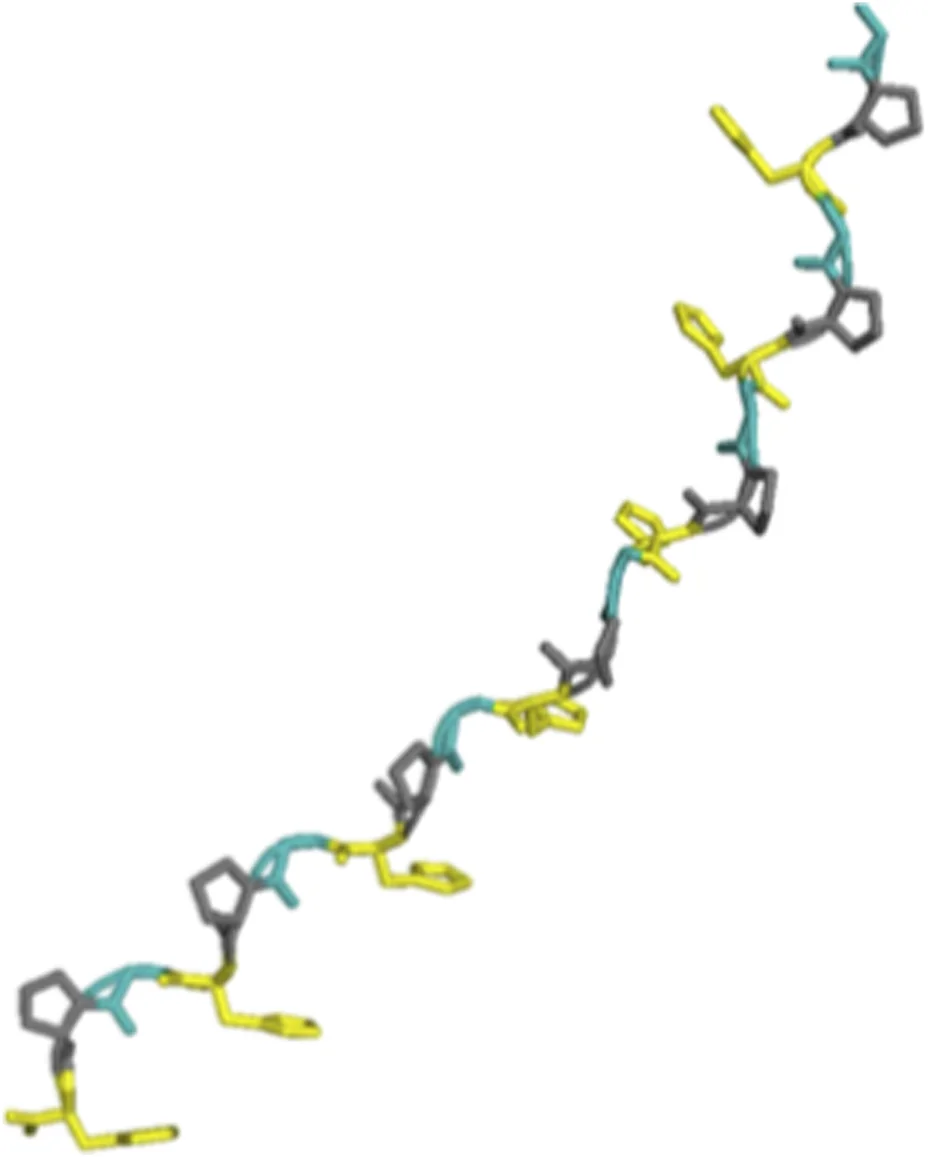
Repeating GPH Subunit, 7 Repeats. Glycine, Proline, and Hydroxyproline are coloured teal, grey and yellow, respectively.

Initial runs of ProteinMPNN using the default weighting parameters showed that hydroxyproline (H) rarely mutated to lysine (K) or other methacrylation-prone residues. To increase sequence diversity and encourage incorporation of methacrylation-prone residues, the sampling temperature was increased to 0.5 and a soft bias of 3.0 was applied to lysine residues. These changes increased the average lysine count from 0.019 to 2.89 residues per sequence, which provides a larger set of candidate peptides for downstream analysis of methacrylation potential and GelE cleavage sites.

The AlphaFold-generated metrics, pLDDT, RMSD and PAE, showed some clear better-performing sequences than others. As shown in Figure 13, higher RMSD models, meaning models that deviate the most structurally from the parent GPH-repeating subunits, have lower confidence (pLDDT) and higher alignment error (PAE).

**FIGURE 13:**
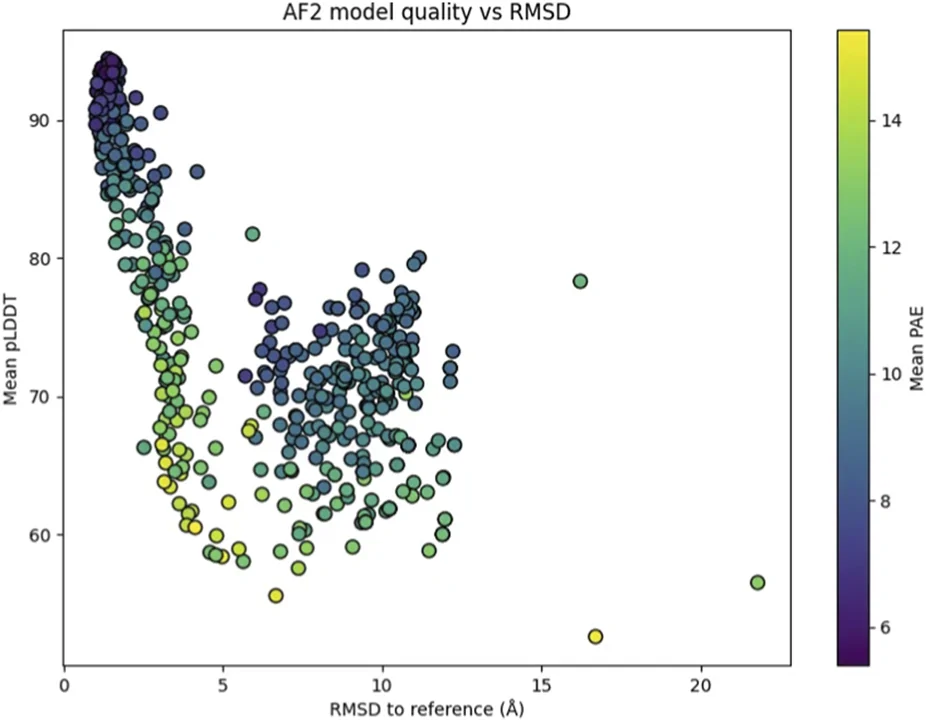
AlphaFold Quality Metrics of Folded MPNN Output Sequences.

An example of a passing subunit is shown in Figure 14a, where there is good alignment with the parent GPH repeating model. This computational workflow resulted in 157 sequences to further analyze. A sample AlphaFold-passing model, which is then re-arranged into a tri-helical structure, is shown in Figure 14b.

**FIGURE 14:**
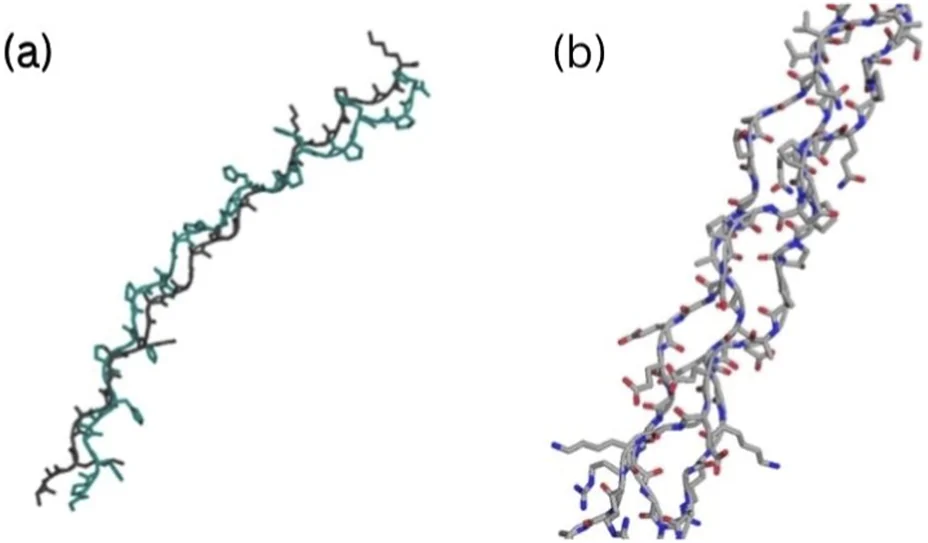
**(a)** Sample AlphaFolded Sequence (Dark Gray) Aligned with Parental GPH Model (Teal). **(b)** Sample Collagen Tri-Helical Unit, Re-assembled from Folded Gelatin Subunit.

After calculating DOM, CLS, and DP on the 157 passing models, the distributions in Figure 15 could be observed, where ‘Composite Score’ can be imagined as a measure of rigidity, computed from (DOM + CLS - DP). It allows for a combined analysis of the balance between cross-linking and degradation.

**FIGURE 15:**
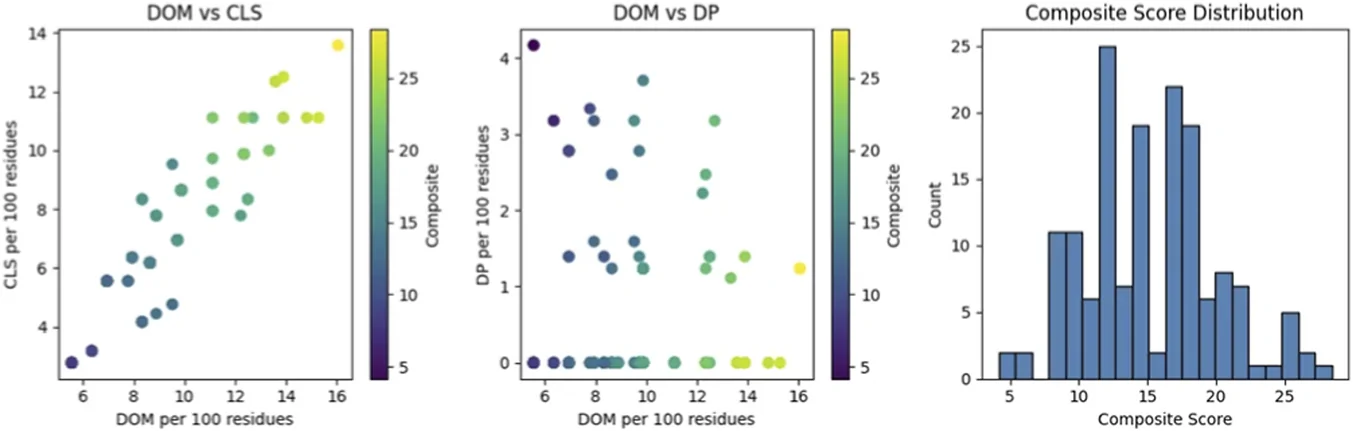
DOM, C2LS, DP and Composite Scores from AlphaFold-Passing Sequences.

The highest composite score, lowest composite score, and median composite score represent candidate rigid, degradable, and balanced options that are all structurally viable. These models are summarized below.

**Table:** 

Proposed scenario	GXY repeats	Sequence	DOM/100	CLS/100	DP/100	Composite
Rigid	9	GAKGPGPPGPKGPPGPGPKGAPGPGPK	16.05	13.58	1.24	28.39
Degradable	8	GAKGPGPPGPKGPPGPGPKGPPGPGAA	5.56	2.78	4.17	4.17
Balanced	9	GPKGPGPGPPGPGAKGPPGAPGPGPK	8.64	6.17	0.00	14.82

## 4 Discussion

This study investigates the potential for a hydrogel-based, enzyme-responsive phage delivery platform to address CAUTIs. By combining wet-lab phage propagation and genetic engineering with computational modelling of hydrogel degradation, phage-bacteria dynamics, and protein design, this study evaluated several key components of the proposed HydroGuard system. While preliminary experimental observations demonstrated the feasibility of individual aspects of the design, the computational models were primarily used to predict system behaviour and guide future optimization rather than to provide experimental validation.

Phage propagation confirmed that lytic *E. coli* phages T2, T4, T7, and λvir can be reliably amplified and characterized, providing the foundation for a GelMA-embedded phage cocktail.

All plasmids and fragments were successfully amplified and gel extracted. Nevertheless, construction of GelE-expressing plasmids provided a BSL-1 surrogate system for studying GelE-mediated enzymatic degradation without directly using pathogenic *E. faecalis*. Although this surrogate system provides a practical and safe approach for evaluating GelE expression, it does not fully reproduce the biological behaviour of native *E. faecalis*, including its regulation of GelE production, biofilm composition, and interactions with bacteriophages. The findings presented here should therefore be interpreted as demonstrating feasibility rather than direct therapeutic performance against *E. faecalis*.

Biofilms were successfully developed after 72 h of cultivation. When using glass slides to produce biofilm they had a thickness of approximately 0.25 mm and 2.6 mg in weight once dried. When testing the product with only the T7 phage injected, limited visible inhibition of *E. coli* was seen, although growth remained similar to the negative control. λ vir alone appeared to produce greater inhibition than T7 alone, although colony growth remained present. Among the conditions evaluated, the phage cocktail showed the greatest apparent reduction in visible bacterial growth. As these observations were qualitative and were not accompanied by quantitative bacterial enumeration, biofilm biomass measurements, or statistical analysis, they should be interpreted as preliminary evidence supporting the feasibility of the HydroGuard concept rather than definitive measurements of antimicrobial efficacy. These observations are consistent with the hypothesis that a cocktail of phages would perform better at combatting biofilm growth than individual phages. Visible reductions in biofilm coverage were also observed, with biofilm only seen in the corners of the slides, furthest away from the injection site, after the 24h incubation. Additionally, small pinprick holes were seen in the hydrogel, suggesting the possibility of GelE-mediated hydrogel degradation.

The computational framework was used to extend these experimental observations across molecular, material, and population scales. Rather than treating the models as separate analyses, they provide complementary descriptions of the proposed HydroGuard mechanism. Agent-based biofilm simulations captured the environmental and spatial conditions surrounding the catheter, while poroelastic diffusion modelling examined how enzymatic degradation alters hydrogel transport and phage availability. The phage-bacteria model then used the predicted phage availability to examine bacterial population dynamics. Protein design simulations further explored collagen-like peptide sequences with properties relevant to controlled degradation. Together, these models provide a multiscale framework linking biofilm conditions to hydrogel degradation, phage release, and bacterial dynamics.

Compared with existing CAUTI prevention strategies, HydroGuard is designed to provide a biologically responsive alternative rather than continuous antimicrobial exposure. Silver-alloy-coated urinary catheters reduce bacterial adhesion but have demonstrated variable effectiveness across clinical studies, while antibiotic-impregnated coatings may contribute to antimicrobial resistance and often rely on passive release mechanisms. In contrast, the proposed HydroGuard system is intended to release bacteriophages only after GelE-mediated degradation of the hydrogel matrix, potentially allowing therapeutic release to occur preferentially in the presence of biofilm-forming bacteria. Although this concept remains to be experimentally validated, the enzyme-responsive release mechanism represents a distinguishing feature compared with conventional antimicrobial coatings. Unlike approaches that provide continuous or passive antimicrobial activity, this design links therapeutic release to a biofilm-associated enzymatic cue. This provides the rationale for using an enzyme-responsive hydrogel as a means of coupling the local bacterial environment to phage availability.

Together, these findings support the feasibility of the proposed design and highlight the potential of enzyme-responsive hydrogels as a dual-function therapeutic platform. This strategy intends to address several key shortcomings of current CAUTI management, including antibiotic overuse, inability to penetrate biofilms, and high recurrence rates. The approach could potentially be adapted, as the modular design of the hydrogel and phage cocktail allows for adaptation against other uropathogens that secrete degradative enzymes.

Beyond its technical novelty, this work holds considerable clinical and societal relevance. A system that releases phages only when GelE-mediated hydrogel degradation occurs has the potential to minimize unnecessary antimicrobial exposure while potentially reducing target biofilms. Reducing the incidence of CAUTIs could lower patient morbidity, prevent recurrent infections, and decrease hospitalizations, particularly among populations that are elderly, chronically ill, or dependent on long-term catheterization. The economic implications are equally important, as CAUTI-related hospitalizations can reach thousands of dollars per case. Effective prevention and treatment would reduce reliance on expensive antibiotics and shorten inpatient stays, alleviating pressures on healthcare systems.

Environmental and biosafety considerations were incorporated into the design with accordance with iGEM regulations. GelMA is biodegradable and safe for disposal and the phages used are lytic and non-toxin-producing. These features may support the future of clinical and environmental safety. With further safety and performance testing, there is potential for clinical translation of this system.

Overall, the work presented here establishes a foundation for further optimization, including validation of gelE-triggered release *in vitro*, and expanded testing in biofilm models. The modularity of this approach also suggests its adaptability to other pathogens that secrete degradative enzymes, providing a broader platform for development of antimicrobial biomaterials and device-associated infection treatments. The present study should therefore be viewed as an early-stage evaluation of an enzyme-responsive antimicrobial biomaterial integrating experimental observations with a multiscale computational framework.

## 5 Conclusion

This study outlines a novel GelE-responsive hydrogel for phage delivery, offering a proposed targeted strategy to prevent and treat CAUTIs. By coupling enzyme-triggered hydrogel degradation with lytic phage release, the system is designed to address some of the limitations of antibiotics in eradicating biofilms. The preliminary experimental observations, together with the computational modelling framework, support the feasibility of the overall design while identifying key areas for future experimental validation.

This work further demonstrates the value of integrating computational biology with biomaterials engineering to guide the rational design of responsive antimicrobial systems. Within the scope of this preliminary study, computational modelling provided predictive insight into hydrogel degradation, phage release, and biofilm interactions while complementing the experimental work.

The multiscale modelling framework developed here provides a predictive platform that may assist in optimizing hydrogel composition, degradation kinetics, and phage release profiles prior to extensive experimental testing.

Such systems-level approaches may facilitate translating complex synthetic biology solutions into clinically viable interventions for device-associated infections when combined with comprehensive experimental validation and material characterization.

Overall, HydroGuard establishes an initial prototype for an enzyme-responsive phage delivery platform and provides a foundation for future studies evaluating its biological performance, material properties, and therapeutic potential.

## Data Availability

Publicly available datasets were analyzed in this study. This data can be found here: https://github.com/igem-waterloo/uwaterloo-igem-2025.
